# Effects of a home-based rehabilitation program in community-dwelling older people after discharge from hospital: A subgroup analysis of a randomized controlled trial

**DOI:** 10.1177/02692155211001672

**Published:** 2021-03-21

**Authors:** Turunen Katri Maria, Aaltonen-Määttä Laura, Portegijs Erja, Rantalainen Timo, Keikkala Sirkka, Kinnunen Marja-Liisa, Sipilä Sarianna, Nikander Riku

**Affiliations:** 1 Gerontology Research Center, and Faculty of Sport and Health Sciences, University of Jyväskylä, Jyväskylä, Finland; 2 Klinik Healthcare Solutions Oy, Helsinki, Finland; 3 Health Centre Hospital, Health Centre of Jyväskylä Cooperation Area, City of Jyväskylä, Finland; 4 Institute of Public Health and Clinical Nutrition, Faculty of Health Sciences, University of Eastern Finland, Kuopio, Finland; 5 Central Hospital of Central Finland, Jyväskylä, Finland; 6 GeroCenter Foundation for Aging Research and Development, Jyväskylä, Finland

**Keywords:** life space, recovery, musculoskeletal disorder, aging

## Abstract

**Objective:**

To examine whether pre-admission community mobility explains the effects of a rehabilitation program on physical performance and activity in older adults recently discharged from hospital.

**Design:**

A secondary analysis of a randomized controlled trial.

**Setting:**

Home and community.

**Participants:**

Community-dwelling adults aged ⩾60 years recovering from a lower limb or back injury, surgery or other disorder who were randomized to a rehabilitation (*n* = 59) or standard care control (*n* = 58) group. They were further classified into subgroups that were not planned a priori: (1) mild, (2) moderate, or (3) severe pre-admission restrictions in community mobility.

**Interventions:**

The 6-month intervention consisted of a motivational interview, goal attainment process, guidance for safe walking, a progressive home exercise program, physical activity counselling, and standard care.

**Measurements:**

Physical performance was measured with the Short Physical Performance Battery and physical activity with accelerometers and self-reports. Data were analysed by generalized estimating equation models with the interactions of intervention, time, and subgroup.

**Results:**

Rehabilitation improved physical performance more in the intervention (*n* = 30) than in the control group (*n* = 28) among participants with moderate mobility restriction: score of the Short Physical Performance Battery was 4.4 ± 2.3 and 4.2 ± 2.2 at baseline, and 7.3 ± 2.6 and 5.8 ± 2.9 at 6 months in the intervention and control group, respectively (mean difference 1.6 points, 95% Confidence Interval 0.2 to 3.1). Rehabilitation did not increase accelerometer-based physical activity in the aforementioned subgroup and did not benefit those with either mild or severe mobility restrictions.

**Conclusions:**

Pre-admission mobility may determine the response to the largely counselling-based rehabilitation program.

## Introduction

The main findings of our randomized controlled trial^1^ showed that the home-based rehabilitation program did not improve physical performance and did not increase the level of physical activity among older people who were recovering from a lower limb or back musculoskeletal injury, surgery or other disorder, over the standard care. However, a similar rehabilitation program that targeted only hip fracture patients was able to restore physical performance among participants who had experienced moderate walking difficulty prior the fracture but not among those who had more severe pre-fracture difficulties in outdoor walking.^2^ This result led to the hypothesis that pre-admission community mobility will determine the response to the rehabilitation in older people after discharge from hospital.

Community mobility refers to the person’s ability to move around in the community and to use public or private transportation.^3^ The Life-Space Assessment^4^ is a valid and reliable measure of community mobility for older patients with gait and balance problems.^5^ It covers both in-home and out-of-home mobility, encompassing a range of activities such as walking, driving and social activities.^4^ Restrictions affecting life space may be interpreted as an early indicator of vulnerability to declining health.^6,7^ An older person may choose to reduce their community mobility in an attempt to compensate for or to accommodate their activity to declining functional abilities.^8^ This potentially leads to greater vulnerability to any adverse events and to a slower and more difficult recovery. In contrast, those with greater community mobility may have more reserves to draw on to assist in their recovery.

The aim of the current subgroup analysis was to investigate whether the effectiveness of the rehabilitation program for older people recently discharged from hospital differed according to their level of pre-admission community mobility. This was assessed in terms of physical activity, physical performance, perceived difficulties in walking and negotiating stairs and fear of falling. This analysis was planned after reporting the primary results of the trial.^1^

## Materials and methods

This is a secondary subgroup analysis of a parallel-group randomized controlled trial (ISRCTN13461584) conducted between February 1, 2016 and February 28, 2018. Ethical approval was received from the research ethics committee of the Central Finland Health Care District (Dnro 3U/2014). A detailed description of the recruitment, design and measurements^9^ and the main results^1^ have been published previously. Briefly, community-dwelling people aged 60 and older were recruited from a health center hospital to which they had been admitted due to a lower limb or back musculoskeletal injury or disorder, including limb or back surgery (e.g. hip fracture, joint replacement, aggravated arthritis), or a fall-related injury. After the hospital discharge and the completion of baseline measurements, they were randomly allocated to either an intervention group (standard care plus the 6-month rehabilitation program) or to the control group (standard care only).

**Figure 1.**
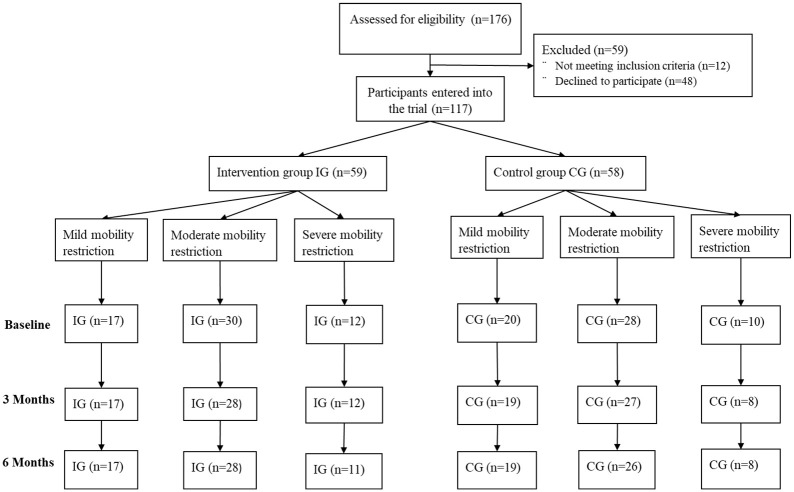
Diagram describing the flow of participants through the trial.

For this subgroup analysis, participants were further categorized into six subgroups based on the group they were originally randomized to and on their self-reported level of community mobility before hospital admission. Community mobility assessment covered the period of time from 1 month prior to their hospital admission. The categories for the subgroups were based on cut-off points used in previous studies^5,7^ and were as follows: (1) mild restrictions: life space assessment score of >56 (*n* = 37), (2) moderate restrictions in mobility: life space assessment score of 31 to 56 (*n* = 58), and (3) severe restrictions in mobility: pre-admission life space assessment score of 0 to 30 (*n* = 22). The flow of participants through the trial is shown in Figure 1.

Physical performance was measured using the Short Physical Performance Battery at baseline, and 3 and 6 months thereafter.^10^ A score is ranging from 0 (worst performance) to 12 (best performance). Perceived difficulties in walking outdoors, in walking 500 m and in negotiating stairs were assessed using a structured questionnaire with the following response categories: (i) able without difficulty, (ii) able with some difficulty, (iii) able with a great deal of difficulty, (iv) unable without the help of another person, and (v) unable to manage even with help. Fear of falling was assessed by the Fall Efficacy Scale–International^11^ at baseline, and at 3 and 6 months thereafter.

Data on physical activity were collected using a single question “Which of the following descriptions best corresponds to your physical activity during the past month?” with seven response categories: (i) mostly resting, mostly lying down, (ii) hardly any activity, mostly sitting, (iii) light physical activity, such as light household tasks, (iv) moderate physical activity for about 3 hours a week: walking longer distances, cycling and domestic work, (v) moderate physical activity for at least 4 hours a week or heavier physical activity for 1 to 2 hours a week, (vi) heavier physical activity or moderate exercise for at least 3 hours a week, and (vii) competitive sports. In addition, a three-dimensional accelerometer was attached to the non-affected anterior thigh line with an adhesive film. Participants were instructed to wear the accelerometer for six consecutive days. We classified physical activity into light, moderate and vigorous intensity, and time spent in physical activity of different intensities was reported in minutes per day. In addition, total activity in minutes per day was included.^1^

A detailed description of the intervention was published previously.^1,9^ All participants received care and rehabilitation according to the usual practice (standard care). In addition to standard care, the intervention group received a home-based individually targeted multi-component rehabilitation program that aimed to promote physical activity and restore mobility. Briefly, seven home visits supervised by a trained physiotherapist were conducted during the 6-month rehabilitation period. These included: (i) setting mobility-related goals, (ii) undergoing a safe walking assessment, (iii) a face-to-face physical activity counselling session, (iv) tailored advice, and (v) coaching for a home exercise program. The home visits were supported by three telephone-coaching calls to increase adherence and facilitate behavioural change.

The exercise program targeted the increase of muscle strength in the lower limbs and improved balance and more fluent walking. Participants were expected to exercise three times a week. The exercise program was checked for exercise type and intensity during each home visit to ensure that the intervention remained appropriate and sufficiently challenging throughout the rehabilitation period. In addition, individual face-to-face physical activity counselling was performed at 3 months after the physiotherapist’s first visit. Motivational interviewing was used to encourage the participants to think and talk about their earlier and current physical activity levels, their interest in returning to previous activities, the possibility of starting a new type of activity or exercise, and how to be active when performing everyday chores. The participant and the physiotherapist both signed a written plan for the participant’s physical activity and evaluated its execution during the final home visit.

### Statistics

Baseline data were expressed as means and standard deviations (continuous variables) or as percentages (categorical variables), and comparisons between groups were made by ANOVA or chi-squared test, as appropriate.

Differences between subgroups at 6 months were analysed by generalized estimating equation models with interactions between group allocation, time, and subgroups regarding pre-admission community mobility. Dependent variables were physical performance, accelerometer-based physical activity, self-reported physical activity, perceived difficulties in negotiating stairs and in walking, and fear of falling. Data were analysed by generalized estimating equation models with the interactions of intervention, time and subgroup. The differences in model-predicted means (with their 95% confidence intervals) for continuous variables between the intervention and control groups were assessed using pairwise-comparisons. The effect size (Hedge’s) between the intervention and control groups was calculated by dividing the difference between the means of each group by the pooled baseline standard deviation (SD) for both groups.^12^ Values between 0.2 and 0.49 were considered a small effect, between 0.5 and 0.79 as a moderate effect and 0.8 and higher as a large effect. Intervention adherence was calculated as (number of exercises performed): (expected number of exercises) × 100%. The analyses were performed with IBM SPSS Statistic Software Version 24 (Chicago, IL).

## Results

Participant mean age was 79.8 years (standard deviation, 8.2), and 86% were women. Baseline characteristics according to study groups are shown in Table 1. The participants in the intervention and control groups in the separate subgroups were similar in terms of baseline characteristics, but among those with mild mobility pre-admission restrictions, controls had a longer stay in hospital (18 vs 9 days, *P* = 0.035) than participants in the intervention group (Table 1).

**Table 1.** Baseline characteristics of participants according to randomized groups and pre-admission community mobility.

Subgroup	Mild mobility restriction		Moderate mobility restriction		Severe mobility restriction	
Outcomes	Intervention (*n* = 17)	Control (*n* = 20)	Intervention (*n* = 30)	Control (*n* = 28)	Intervention (*n* = 12)	Control (*n* = 10)
Age, mean (SD), years	76 (8)	77 (8)	82 (9)	81 (8)	80 (6)	80 (9)
Women, *n* (%)	13 (77)	16 (80)	29 (97)	25 (89)	8 (67)	9 (90)
Years of education, mean (SD)	11 (5)	10 (4)	10 (5)	9 (3)	10 (3)	8 (2)
MMSE points, mean (SD)	27 (2)	27 (2)	26 (3)	25 (3)	26 (2)	25 (2)
Living alone, *n* (%)	11 (65)	16 (80)	19 (63)	17 (61)	8 (67)	7 (70)
Body Mass Index, mean (SD), kg/m^2^	26 (6)	27 (4)	28 (4)	29 (6)	27 (4)	25 (4)
Poor self-rated health, *n* (%)	5 (30)	5 (25)	19 (63)	15 (54)	8 (67)	3 (30)
Number of chronic diseases, mean (SD)	2 (2)	2 (2)	3 (2)	4 (2)	3 (1)	3 (2)
Reasons for hospitalization, *n* (%)						
Traumatic fracture	7 (41)	8 (40)	11 (37)	11 (39)	5 (41)	5 (50)
Intensified pain in back or lower extremity (i.e. following falling)	3 (18)	4 (20)	5 (17)	7 (25)	2 (17)	1 (10)
Intended joint replacement	7 (41)	6 (30)	13 (43)	9 (32)	3 (25)	3 (30)
Intended back surgery	0	2 (10)	1 (3)	1 (4)	2 (17)	1 (10)
Length of hospital stay, days, mean (SD)	9 (7)	18 (16)	15 (13)	19 (20)	18 (15)	16 (8)
Interference of pain, mean (SD)	5 (2)	3 (3)	4 (3)	4 (3)	5 (3)	5 (2)
Mood, CES-D points, mean (SD)	13 (8)	9 (8)	13 (10)	17 (11)	18 (13)	17 (11)
Fear of falling, FES-I score, mean (SD)	38 (12)	34 (12)	39 (10)	41 (11)	44 (13)	43 (9)
Accelerometer-based physical activity total, minutes/day, mean (SD)	215 (108)	206 (96)	175 (117)	175 (103)	149 (70)	120 (83)
Baseline SPPB, score, mean (SD)	6 (2)	6 (3)	4 (2)	4 (2)	4 (2)	4 (2)
Life space mobility (LSA) score prior to hospitalization, mean (SD)	76 (13)	74 (13)	42 (8)	42 (8)	22 (9)	25 (5)
Stairs negotiation, *n* (%)						
No difficulties	1 (6)	3 (15)	1 (3)	5 (18)	1 (8)	2 (20)
Minor difficulties	9 (53)	7 (35)	6 (20)	2 (7)	4 (33)	3 (30)
Major difficulties	2 (12)	3 (15)	3 (10)	6 (21)	1 (8)	1 (10)
Manage only with help	1 (6)	5 (25)	11 (37)	7 (25)	2 (18)	2 (20)
Unable to manage even with help	4 (24)	2 (10)	9 (30)	8 (29)	4 (33)	2 (20)
Self-reported level of physical activity prior to hospitalization, *n* (%)						
Inactivity	0	0	4 (13)	4 (14)	5 (42)	1 (10)
Low level activity	1 (6)	4 (20)	14 (47)	13 (46)	7 (58)	6 (60)
Medium to high level activity	16 (94)	16 (80)	12 (40)	11 (39)	0	3 (30)

SD: standard deviation; SPPB: short physical performance battery; MMSE: mini mental state examination; CES-D: Center for Epidemiologic Studies Depression Scale; FES-I: Fall Efficacy Scale-International; LSA: University of Alabama at Birmingham study of aging life space assessment.

Adherence to the physical exercise program varied according to the pre-admission community mobility: with 82% (*n* = 14) adherence to the planned physical exercise program by participants with mild pre-admission mobility restriction, 53% (*n* = 16) by those with moderate restriction, and 42% (*n* = 5) by those with severe restriction.

The mean score of the Short Physical Performance Battery increased 27% more in the intervention compared to the control group among those with moderate pre-admission mobility restriction (interaction *P*-value = 0.026; Table 2; Supplemental Figure S1). Among participants with moderate community mobility restriction, the intervention reduced perceived difficulties in negotiating stairs (interaction *P* = 0.007), walking outdoors (*P* = 0.037), and walking 500 m (*P* = 0.035) relative to the standard care only at 6 months post intervention (Supplemental Table S1). Similarly, the intervention was effective in reducing fear of falling among those with moderate pre-admission mobility restriction (interaction *P* = 0.002). Among the other subgroups, the intervention was not superior to the standard care in terms of physical performance, perceived stairs-negotiation, walking outcomes or fear of falling (Table 2).

**Table 2.** Intervention effects (baseline to post intervention) on physical performance, accelerometer-derived physical activity, and fear falling in subgroups.

Subgroup	Participants with mild pre-admission mobility restriction						Mean difference IG − CG (95% CI)	*P*-value	Effect size
Outcome	Intervention (mean ± SD)			Control (mean ± SD)					
	Baseline (*n* = 17)	Three months (*n* = 17)	Six months (*n* = 17)	Baseline (*n* = 20)	Three months (*n* = 19)	Six months (*n* = 19)			
SPPB (score)	6.0 ± 2.4	8.6 ± 2.1	8.9 ± 2.6	5.8 ± 2.6	8.4 ± 2.4	8.9 ± 3.0	−0.10 (−1.87 to 1.68)	0.916	−0.080
Total physical activity (minutes/day)	153 ± 81	209 ± 75	238 ± 77	143 ± 70	210 ± 77	198 ± 77	37.61 (−7.66 to 82.89)	0.103	0.390
Light activity, (minutes/day)	119 ± 46	136 ± 43	153 ± 41	117 ± 51	128 ± 38	121 ± 36	30.40 (5.65 to 55.15)	0.016	0.611
Moderate to vigorous activity (minutes/day)	34 ± 50	74 ± 45	85 ± 49	26 ± 26	82 ± 52	77 ± 45	9.95 (−4.42 to 24.33)	0.175	0.013
FES-I (score)	37 ± 12	28 ± 8	29 ± 9	34 ± 12	26 ± 7	26 ± 9	2.09 (−3.68 to 7.88)	0.477	0.083
Subgroup	Participants with moderate pre-admission mobility restriction								
	Baseline (*n* = 30)	Three months (*n* = 28)	Six months (*n* = 28)	Baseline (*n* = 28)	Three months (*n* = 27)	Six months (*n* = 26)			
SPPB (score)	4.4 ± 2.3	6.4 ± 2.1	7.3 ± 2.6	4.2 ± 2.2	5.3 ± 2.9	5.8 ± 2.9	1.62 (0.19 to 3.06)	0.027	0.569
Total physical activity (minutes/day)	121 ± 83	159 ± 64	152 ± 57	120 ± 70	139 ± 70	140 ± 59	12.64 (−18.71 to 43.99)	0.429	0.141
Light activity (minutes/day)	104 ± 62	118 ± 44	113 ± 38	100 ± 49	109 ± 48	111 ± 44	2.98 (−19.10 to 25.06)	0.791	−0.035
Moderate to vigorous activity (minutes/day)	18 ± 31	41 ± 31	39 ± 30	19 ± 28	30 ± 33	29 ± 25	10.0 (−4.42 to 24.33)	0.175	0.367
FES-I (score)	39 ± 10	29 ± 9	28 ± 9	41 ± 11	33 ± 11	36 ± 9	−7.81 (−12.75 to −2.87	0.002	−0.564
Subgroup	Participants with severe pre-admission mobility restriction								
	Baseline (*n* = 12)	Three months (*n* = 11)	Six months (*n* = 11)	Baseline (*n* = 10)	Three months (*n* = 9)	Six months (*n* = 8)			
SPPB (score)	3.7 ± 2.5	4.8 ± 3.1	4.9 ± 3.3	3.7 ± 2.5	4.7 ± 3.1	5.9 ± 4.2	−0.70 (−3.72 to 2.33)	0.652	−0.385
Total physical activity (minutes/day)	104 ± 50	95 ± 41	95 ± 48	80 ± 55	122 ± 71	126 ± 82	−30.37 (−85.52 to 24.76)	0.280	−0.932
Light activity (minutes/day)	93 ± 47	78 ± 32	75 ± 30	75 ± 53	97 ± 45	93 ± 51	−18.97 (−54.55 to 16.62)	0.296	−0.696
Moderate to vigorous activity (minutes/day)	11 ± 9	17 ± 16	19 ± 21	4 ± 5	26 ± 30	33 ± 33	−11.32 (−34.50 to 11.86)	0.338	−2.705
FES-I (score)	44 ± 13	42 ± 14	35 ± 8	43 ± 9	31 ± 14	31 ± 10	4.00 (−3.51 to 11.52)	0.297	0.254

SD: standard deviation; IG: intervention group; CG: control group; SPPB: short physical performance battery; FES-I: Fall Efficacy Scale-International.

No between-group (intervention vs control) differences were observed in total daily time spent in physical activity, or in time spent in mild, moderate, or vigorous physical activity in the three subgroups studied (Table 2). However, we did observe a statistically significant increase in self-reported physical activity in the intervention group compared to the controls among those with moderate pre-admission mobility restriction (interaction *P* = 0.013, Supplemental Table S1).

## Discussion

As reported previously, physical performance of older people who were recovering from a lower limb or back musculoskeletal injury, surgery, or other disorder was unaffected by the intervention.^1^ However, when considering pre-admission community mobility, rehabilitation improved physical performance more in the intervention group than in the control group among participants with moderate community mobility restriction. In detail, Short Physical Performance improved clinically meaningfully from 0.4 to 1.5 points, which is enough to reduce incident mobility disability among older adults.^13^ In addition, perceived difficulty in negotiating stairs and in walking reduced in the intervention group compared to the control group among participants who had experienced moderate pre-admission mobility restrictions. Among participants with severe pre-admission restrictions in community mobility or, on the other hand, among those who were fittest prior to hospitalization, the intervention did not appear to be superior to standard care in terms of physical performance, or self-reported walking outcomes.

Our results are consistent with previous studies showing that among hip fracture patients,^2,14^ people with sufficient physical capacity and mobility prior to hospitalization can attain broader intervention-induced mobility benefits. Older people with moderate restrictions in their life space seem to have the potential to improve their physical performance by following a home-based rehabilitation program. They may have been experiencing a slow worsening of health problems over the months pre-admission, which resulted in their moderate life space restrictions. However, having received appropriate treatment during their hospital stay, they probably have the resources to recover and the motivation to work for their recovery. Their mobility was not too restricted for independent physical training, but they needed guidance or even assistance to go outdoors from their homes in the beginning of the rehabilitation program. Thus, a supervised home-based program was the best match for their needs and characteristics. A previous study by Loyd et al.^15^ also showed that participants with a moderate mean score (43 points) for pre-admission community mobility were able to improve their walking to a greater extent than those who had higher scores in community mobility prior to hospitalization. It is important as older adults with a community mobility score of ⩽52.3 are particularly at risk for developing functional decline over a 2-year follow-up.^6^

In our study, participants with moderately restricted community mobility did not differ from participants with greater restriction before hospitalization in terms of age, cognition or reasons for hospitalization, but they had fewer problems related to mobility and they were more physically active. As indicated by the relatively low compliance, it is likely that the intervention was too demanding for participants with severe mobility restrictions. They possibly need a comprehensive geriatric assessment and a more supervised reablement service, planned by a multi-professional team. On the other hand, the participants with fewer restrictions in their life space before hospitalization probably experienced only a temporary mobility loss, and they were able to recover quickly in months following the hospitalization due to their better physiological reserves. For them, the home-based rehabilitation program did not offer additional benefits.

The strengths of this study are the validated and widely applied methods that were used to measure the outcomes. The findings from both the performance-based test and self-reported outcomes support the positive effects of the intervention on the mobility of older people who had moderate restrictions in their community mobility prior to hospitalization. The study population consisted of older people with a wide range of functional capacities, as is normal in an older population. Thus, this study provides important “real-world” information. Another strength of the study is that the intervention involved strategies tested earlier in large trials with older people.

The study also has major limitations. The sample consisted mostly of women, and although they represent the majority of the older population, males were probably under-represented. We report here a post-hoc analysis with subgroups that were not planned before the study started, and there is thus a lack of statistical power among these relatively small subgroups. The community mobility cut-points that were used to define the subgroups were, however, based on prior theory and earlier research.^5,7^ The analyses were not adjusted for multiple testing, and thus the risk of Type 1 error exists.

This secondary analysis of the randomized controlled trial generates the hypothesis that a home-based, personalized, rehabilitation program restores physical performance, and walking abilities in older people with moderate restrictions in community mobility prior to hospital admission. This study is only exploratory, and future randomized controlled trials are required to test the suggested hypothesis and confirm the findings. This study does not provide sufficient evidence on which to base clinical decisions. However, the results suggest the importance of evaluating pre-existing mobility and functional abilities during hospitalization and immediately after discharge as this will make it possible to tailor appropriate interventions from which the individuals will be most likely to benefit. This could prevent further disability and promote functional independence, thereby relieving both the individual and the health system burden.

**Clinical messages**

- Adherence and response to home-based rehabilitation varied according to pre-admission community mobility scores among older people who are recovering from lower limb or back injury, surgery or disorder.
- This study suggests that older people with moderate pre-admission mobility restrictions benefit from home-based rehabilitation program but further larger trials are required to confirm the results.

## Supplemental Material

sj-pdf-1-cre-10.1177_02692155211001672 – Supplemental material for Effects of a home-based rehabilitation program in community-dwelling older people after discharge from hospital: A subgroup analysis of a randomized controlled trial Click here for additional data file. Supplemental material, sj-pdf-1-cre-10.1177_02692155211001672 for Effects of a home-based rehabilitation program in community-dwelling older people after discharge from hospital: A subgroup analysis of a randomized controlled trial by Turunen Katri Maria, Aaltonen-Määttä Laura, Portegijs Erja, Rantalainen Timo, Keikkala Sirkka, Kinnunen Marja-Liisa, Sipilä Sarianna and Nikander Riku in Clinical Rehabilitation
