# Supplementary material for: Effects of a home-based rehabilitation program in community-dwelling older people after discharge from hospital: A subgroup analysis of a randomized controlled trial
Source: Clin Rehabil. 2021 Mar 21;35(9):1257–65. doi: 10.1177/02692155211001672 (PMC8369904; doi:10.1177/02692155211001672)
Supplement: sj-pdf-1-cre-10.1177_02692155211001672 – Supplemental material for Effects of a home-based rehabilitation program in community-dwelling older people after discharge from hospital: A subgroup analysis of a randomized controlled trial [file sj-pdf-1-cre-10.1177_02692155211001672.pdf]

Table S1. Changes in self-reported level of physical activity, perceived difficulties in negotiating stairs, walking outdoors, and walking 500 meters; proportion of participants and response categories for the intervention and control groups at pre-admission, baseline and at 3 and 6 months post-intervention.

Subgroup ►	Participants with mild pre-admission mobility restriction								
	Intervention				Control				
	Pre-adm, n = 17	BL, n = 17	3 Mo, n = 17	6 Mo, n = 17	Pre-adm, n = 20	BL, n = 20	3 Mo, n = 18	6 Mo, n = 19	Group x Time interaction p-value
Self-reported physical activity, n (%)									0.446
Mostly lying down	0	0		0	0	1 (5)	0	0	
Mostly sitting	0	4 (23)	2 (12)	0	0	5 (25)	0	0	
Low level activity	1 (6)	13 (77)	4 (24)	4 (24)	4 (20)	13 (65)	4 (22)	5 (26)	
Medium level activity, ≥ 3 hrs/week	7 (41)	0	3 (18)	3 (18)	7 (35)	1 (5)	3 (17)	5 (26)	
Medium level activity, ≥ 4 hrs/week	6 (35)	0	6 (35)	5 (29)	6 (30)	0	10 (56)	5 (26)	
High level activity, exercise	3 (18)	0	2 (12)	5 (29)	3 (15)	0	1 (6)	4 (21)	

Negotiating stairs, n (%)									0.826
No difficulties	7 (41)	1 (6)	8 (47)	11 (64)	9 (45)	3 (15)	9 (50)	7 (37)	
Minor difficulties	4 (23)	9 (52)	6 (35)	3 (18)	8 (40)	7 (35)	8 (44)	11 (58)	
Major difficulties	5 (29)	2 (12)	3 (18)	3 (18)	3 (15)	3 (15)	1 (6)	1 (5)	
Only with help of another person	0	1 (6)	0	0	0	5 (25)	0	0	
Unable even with help	1 (6)	4 (24)	0	0	0	2 (10)	0	0	
Walking outside, n (%)									0.181
No difficulties	7 (41)	1 (6)	2 (12)	6 (35)	10 (50)	3 (15)	9 (50)	12 (63)	
Minor difficulties	7 (41)	9 (53)	13 (76)	9 (53)	8 (40)	9 (45)	9 (50)	7 (37)	
Major difficulties	2 (12)	2 (12)	2 (12)	2 (12)	2 (10)	5 (25)	0	0	
Only with help of another person	0	5 (29)	0	0	0	3 (15)	0	0	
Unable even with help	1 (5)	0	0	0	0	0	0	0	
Walking 500 meters, n (%)									0.555
No difficulties	7 (41)	2 (12)	6 (35)	9 (53)	11 (55)	3 (15)	11 (61)	13 (68)	
Minor difficulties	8 (47)	5 (29)	10 (59)	5 (29)	5 (25)	7 (35)	6 (33)	6 (32)	
Major difficulties	1 (6)	3 (18)	1 (6)	2 (12)	1 (5)	4 (20)	1 (6)	0	
Only with help of another person	0	1 (6)	0	0	0	2 (10)	0	0	

Unable even with help	1 (6)	6 (35)	0	1 (6)	3 (15)	4 (20)	0	0	
Subgroup ►	Participants with moderate pre-admission mobility restriction								
Self-reported physical activity, n (%)									0.013
Mostly lying down	0	4 (13)	1 (3)	0	0	3 (10)	1 (4)	1 (4)	
Mostly sitting	4 (13)	11 (37)	3 (11)	1 (3)	4 (14)	10 (37)	4 (15)	2 (7)	
Low level activity	14 (47)	15 (50)	15 (54)	15 (54)	13 (46)	14 (50)	16 (59)	15 (58)	
Medium level activity, ≥ 3 hrs/week	9 (30)	0	5 (18)	7 (25)	8 (29)	0	3 (11)	3 (12)	
Medium level activity, ≥ 4 hrs/week	3 (10)	0	4 (14)	3 (11)	3 (11)	1 (3)	3 (11)	4 (15)	
High level activity, exercise	0	0	0	2 (7)	0		0	1 (4)	
Negotiating stairs, n (%)									0.007
No difficulties	7 (23)	1 (3)	10 (36)	10 (36)	3 (11)	5 (18)	6 (22)	2 (7)	
Minor difficulties	9 (30)	6 (20)	13 (47)	10 (36)	11 (39)	2 (7)	8 (30)	12 (48)	
Major difficulties	10 (33)	3 (10)	2 (7)	4 (14)	8 (29)	6 (21)	4 (15)	6 (23)	
Only with help of another person	2 (7)	11 (37)	2 (7)	2 (7)	1 (3)	7 (25)	3 (11)	2 (7)	
Unable even with help	2 (7)	9 (30)	1 (3)	2 (7)	5 (18)	8 (29)	6 (22)	4 (15)	
Walking outside, n (%)									0.037

No difficulties	7 (23)	1 (3)	9 (33)	11 (40)	5 (18)	2 (7)	3 (11)	3 (12)	
Minor difficulties	12 (40)	10 (34)	14 (50)	14 (50)	14 (50)	5 (18)	16 (59)	15 (58)	
Major difficulties	9 (30)	4 (13)	3 (11)	1 (3)	8 (29)	8 (29)	4 (15)	4 (15)	
Only with help of another person	2 (7)	12 (40)	1 (3)	2 (7)	1 (3)	13 (46)	4 (15)	4 (15)	
Unable even with help	0	3 (10)	1 (3)	0	0	0	0	0	
Walking 500 meters, n (%)									
No difficulties	7 (23)	0	6 (22)	9 (32)	3 (11)	2 (7)	4 (14)	3 (12)	0.035
Minor difficulties	8 (27)	4 (13)	15 (54)	12 (43)	9 (32)	3 (10)	10 (37)	12 (46)	
Major difficulties	7 (23)	4 (13)	4 (14)	3 (11)	7 (25)	5 (18)	5 (19)	2 (7)	
Only with help of another person	3 (10)	0	1 (3)	2 (7)	0	1 (3)	3 (11)	3 (12)	
Unable even with help	5 (17)	22 (73)	2 (7)	2 (7)	9 (32)	17 (62)	5 (19)	6 (23)	
Subgroup ►	Participants with severe pre-admission mobility restriction								
Self-reported physical activity, n (%)									0.067
Mostly lying down	1 (8)	2 (17)	1 (8)	0	0	2 (20)	0	2 (25)	
Mostly sitting	4 (33)	4 (33)	2 (17)	3 (27)	1 (10)	5 (50)	2 (24)	0	
Low level activity	7 (58)	6 (50)	6 (50)	5 (46)	6 (60)	3 (30)	1 (13)	2 (25)	

Medium level activity, ≥ 3 hrs/week	0	0	3 (25)	1 (9)	3 (30)	0	4 (50)	3 (37)	
Medium level activity, ≥ 4 hrs/week	0	0	0	1 (9)	0	0	1 (13)	1 (13)	
High level activity, exercise	0	0	0	1 (9)	0	0	0	0	
Negotiating stairs, n (%)									0.610
No difficulties	1 (8)	1 (8)	2 (17)	2 (18)	2 (20)	2 (20)	3 (38)	0	
Minor difficulties	1 (8)	4 (33)	4 (33)	3 (27)	1 (10)	3 (30)	2 (25)	4 (50)	
Major difficulties	3 (25)	1 (8)	0	2 (18)	3 (30)	1 (10)	0	0	
Only with help of another person	2 (17)	2 (17)	2 (17)	1 (10)	3 (30)	2 (20)	2 (25)	2 (25)	
Unable even with help	5 (42)	4 (33)	4 (33)	3 (27)	1 (10)	2 (20)	1 (12)	2 (25)	
Walking outside, n (%)									
No difficulties	1 (8)	0	1 (8)	0	1 (10)	1 (10)	2 (25)	6 (75)	0.260
Minor difficulties	3 (25)	4 (33)	6 (50)	5 (46)	2 (20)	1 (10)	3 (38)	0	
Major difficulties	5 (42)	4 (33)	1 (8)	2 (18)	4 (40)	2 (20)	1 (12)	1 (13)	
Only with help of another person	1 (8)	2 (17)	4 (33)	4 (36)	3 (30)	5 (50)	2 (25)	1 (13)	
Unable even with help	2 (17)	2 (17)	0	0	0	1 (10)	0	0	
Walking 500 meters, n (%)									0.421
No difficulties	1 (8)	0	2 (17)	0	0	1 (10)	2 (25)	2 (25)	

Minor difficulties	2 (17)	1 (8)	3 (25)	4 (37)	3 (30)	0	2 (25)	4 (50)	
Major difficulties	2 (17)	4 (34)	2 (17)	2 (18)	4 (40)	1 (10)	1 (12)	0	
Only with help of another person	0	0	2 (17)	2 (18)	2 (20)	1 (10)	0	0	
Unable even with help	7 (58)	7 (58)	3 (25)	3 (27)	1 (10)	7 (70)	3 (38)	2 (25)	

Pre-adm=pre-admission, BL=baseline, Mo=months, hrs=hours.

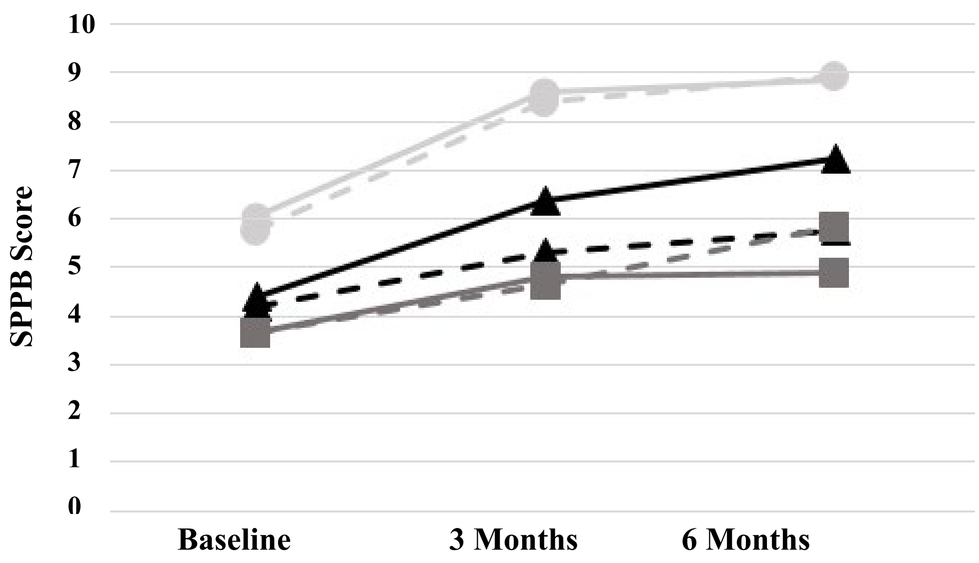

Figure S1: Short Physical Performance Battery Score (SPPB) by treatment arm and pre-admission mobility. Solid line= intervention group, dashed line= control group, ⬤ = participants with mild preadmission mobility restriction, ▲= participants with moderate preadmission mobility restriction, ■ = participants with severe preadmission mobility restriction.
